# The Feasibility of Providing Remote Functional Family Therapy with Adolescents During the COVID-19 Pandemic: A Mixed-Method Study

**DOI:** 10.1007/s10566-022-09692-y

**Published:** 2022-05-02

**Authors:** Aurelie M. C. Lange, Sajid Humayun, Tom Jefford

**Affiliations:** 1 Family Psychology Mutual CIC, Huntingdon, UK; 2 School of Human Science, University of Greenwich, London, UK

**Keywords:** Videoconferencing, Systemic therapy, Covid-19, Feasibility, Mixed-method

## Abstract

**Background:**

Due to the recent COVID-19 pandemic, mental health care has largely transferred its services to online platforms, using videoconferencing (VC) or teletherapy. Within the field of family therapy, however, there is little evidence on the feasibility of using VC, especially when working with whole families at the edge of care.

**Objective:**

This study investigated the feasibility of remote Functional Family Therapy (FFT), using a mixed-method approach.

**Method:**

Study 1 consisted of semi-structured interviews with 23 FFT professionals (18 female) about their experience of providing remote FFT during the COVID-19 pandemic. Study 2 included monitoring data of 209 FFT clients (46% female, *M*_*age*_ = 14.00) who participated in FFT during the pandemic. We compared families who received mainly in-person, mainly remote or a mix of remote and in-person on client-reported alliance, drop-out, therapist-rated outcomes, and treatment intensity using MANCOVA’s and chi-square tests.

**Results:**

In Study 1 two themes emerged around experienced challenges, namely ‘Feeling in control’ and ‘Engagement and alliance’. Two other themes emerged around adaptations, namely ‘Being more on top’ and ‘Connecting in different ways’. In Study 2, we found that the therapeutic alliance was not related to using VC. Also, families had less between-session contact during the Engagement and Motivation Phase when receiving mainly VC, but had more sessions and longer therapy when receiving a mix of in-person and remote therapy.

**Conclusions:**

The current study suggests that providing systemic family teletherapy to families on the edge of care is feasible. Further development of systemic family teletherapy is warranted.

**Supplementary Information:**

The online version contains supplementary material available at 10.1007/s10566-022-09692-y.

## Introduction

With the global spread of COVID-19, the delivery of mental health care had to be fundamentally reorganised. In March 2019, the United Kingdom (UK), together with many other countries, went into a national lockdown, requiring everyone to work from home. For many mental health care providers, this meant transferring their service to online platforms and providing therapy through videoconferencing (VC), i.e. teletherapy. For the majority of mental health providers, providing teletherapy is completely new (Békés & Aafjes-van Doorn, 2020) and within the profession of family therapy and couple therapy, experience with teletherapy is even more limited (Mc Kenny et al., 2021).

Yet, teletherapy has the potential to increase access to therapy and reduce costs (Bennett et al., 2020; Massoudi et al., 2019). Teletherapy can unlock access to care for clients living in remote areas (Bennett et al., 2020), such as access to specialised or intensive family interventions, which are often mainly provided in urban areas. It can also increase accessibility for specific members of the family to participate in therapy (Burgoyne & Cohn, 2020; Heiden-Rootes et al., 2021), with fathers and teenagers being potentially more likely to engage (Mc Kenny et al., 2021).

The pandemic therefore provides a unique opportunity to increase our understanding of both the potential and the barriers of conducting teletherapy with families. This mixed-method study contributes to this by analysing qualitative and quantitative data from family therapists and families nine months into the pandemic.

### Videoconferencing in Systemic Family Therapy

Evidence about the effectiveness of VC for systemic family therapy is scarce, however, fuelled by the pandemic, a meta-analysis and a systematic review have emerged in the past two years (De Boer et al., 2021; McLean et al., 2021). Both studies found that teletherapy outcomes are comparable to in-person therapy and that teletherapy for families and for couples is feasible and acceptable.

Most of these studies, however, focussed on couples or sessions with the referred child and one parent, where clients were self-referred and presented mild problems (De Boer et al., 2021; Helps & Le Coyte Grinney, 2021). Much less research is available about providing systemic family therapy to whole families, especially hard-to-engage families on the edge of care with severe problems and potentially presenting high risks (e.g., family violence). Family therapy may be particularly beneficial for these families, if traditional barriers to engagement and attendance can be overcome, but they may present more challenges when being treated with VC.

The current evidence around VC may therefore not apply to this specific population. This study addresses the feasibility of treating this high-risk and difficult-to-engage population using VC. Rolling out VC with this population without evidence of its feasibility may create unsafe situations; it could cause harm to clients, increase drop-out or lead to burn-out from therapists, thereby reducing treatment effectiveness and increasing costs.

### Using VC with Families on the Edge of Care

Working with families on the edge of care using VC may present some specific challenges. Firstly, using VC with whole families may be more challenging. Therapists cannot see many of the non-verbal cues and nonverbal interactions between family members, which is a central aspect of systemic family therapy that needs to be attended to (Heiden-Rootes et al., 2021). Involving young children and screen-shy adolescents has also been mentioned as challenging. Younger children may not have the concentration span to sit in front of a screen and usually require a more interactive approach. Adolescents have been reported to be very self-aware in front of a camera, resulting in them not wanting to be seen on a screen (Burgoyne & Cohn, 2020; Mc Kenny et al., 2021). Involving younger children or adolescents is thus challenging on its own and potentially even more challenging when engaging multiple children at the same time. It may be a common misconception that the digitally aware younger generation are ideally suited to online engagement.

Secondly, there may be challenges around engaging these families when using VC. Many families on the edge of care struggle with a range of problems within the family system and have been involved with numerous care professionals for a number of years. This may result in mistrust towards new professionals, lack of motivation for additional treatments, and a lack of hope of any successful outcome (Deković & Bodden, 2019; Escudero & Friedlander, 2017; McLeod et al., 2016). Professionals may need to work considerably harder to engage all family members, for example by visiting families in their homes, sometimes multiple times a week (Visscher et al., 2020). This may be much harder when using VC. Therapists also need to balance alliances with all family members; having a strong therapeutic alliance or working relationship with one member of the family may be detrimental for the therapeutic process if this leads to weak alliances with other members (i.e., a split alliance) (Friedlander et al., 2018; Muñiz de la Peña et al., 2009). Although this is not specific to VC, this may be more challenging when using VC, for example, if not all family members are visible on the screen.

Finally, managing risks may be especially problematic when using VC. Previous research has suggested that it may be harder to assess and manage interactions between family members through a screen. This may create risky situations and anxiety in therapists if interactions evolve into conflicts (Burgoyne & Cohn, 2020; McLean et al., 2021; Springer et al., 2020). This is a risk within any family or couple therapy but may be more likely to happen or have more severe consequences if there is a history of violence in the family.

### Current Study

The current study explored the feasibility of providing Functional Family Therapy (FFT), a relational, systemic therapy for families on the edge of care, through VC. Families are commonly referred by social workers and may not be initially motivated for therapy. FFT takes a strength-based approach and therapists need to be persistent to create engagement. Therapists usually provide weekly in-home sessions for a period of three to five months, whereby all family members are expected to attend the sessions (Robbins et al., 2016). FFT thus provides a unique opportunity to study the feasibility of providing whole-family systemic teletherapy to high-risk and difficult-to-engage families. Feasibility is defined as the extent to which an innovation (e.g., VC) can be successfully used in a specific setting (e.g., with families on the edge of care) and involves an evaluation of potential barriers (Rolland et al., 2021).

We opportunistically designed a sequential mixed-method approach aiming for convergence and complementarity (Palinkas et al., 2011), using qualitative interviews (Study A) followed by quantitative analyses of routinely collected monitoring data (Study B). Convergence was achieved by testing hypotheses from the qualitative study, thus providing triangulation. The unique strengths of each design further allowed for findings to complement one another, whereby the qualitative study focused on barriers and adaptations to using VC and the quantitative study focused on measuring the effect of VC on alliance, treatment length, drop-out and outcomes. The aims of the study were to (1) determine if the delivery of FFT online was adversely affected through this method of delivery, (2) to understand barriers and adaptations to the online delivery of FFT, and (3) to understand if a fully online or blended delivery of FFT (partially online) is good enough to become an acceptable model of delivery post pandemic. We developed additional specific hypotheses for Study B based on the findings from Study A.

## Study A

### Method

#### Design and Procedure

A semi-structured one-hour interview was devised and piloted with four FFT professionals in December 2020. After making minor adaptations, another 19 interviews were conducted in February 2021. All participants participated in a single interview. Interview questions related to the following topics (see Supplementary materials for full interview format): (1) whether and how the use of VC affected FFT delivery, (2) whether and how VC affected assessment and management of risks, (3) whether and how the pandemic affected professionals personally, (4) the fit of VC to families and professionals, and (5) future use of VC. The topics were discussed in a conversational style and did not necessarily follow the order of the interview format or adhere to the specific formulation from the interview format. All interviews took place online through a one-to-one video-call. Interviews were transcribed using transcription software and checked for accuracy by the first author. Field notes were made on an ad hoc basis throughout the study. Interviewees did not revisit their transcripts for respondent validation. Findings were presented to the teams at the end of the project.

The majority of the interviews (sixteen, including the four pilot interviews) were conducted by the first author (interviewer 1), a female PhD researcher with experience in the field of evidence-based systemic interventions and some prior knowledge of the scientific literature on teletherapy, but no clinical experience. Seven interviews were conducted by a female FFT therapist (interviewer 2) who conducted the interviews as part of her Master’s degree in Systemic Family Therapy. Both interviewers were employed by Family Psychology Mutual CIC (FPM), one of the participating institutions delivering FFT. The first interviewer presented the research to the FFT teams prior to inviting therapists for an interview. Participants were told the aim was to better understand their experience around providing FFT using VC to inform the potential future provision of blended or fully remote FFT. The second interviewer did not know the participants prior to interviewing them. Ethical approval was granted by the University Research Ethics Committee of the University of Greenwich. Informed consent was acquired prior to the interviews. The first and third author were employed during this study by one of the organisations providing Functional Family Therapy studied in this research.

#### Participants

All therapists, supervisors and team managers working at the two participating institutions (approximately 25–30) were invited for an interview through e-mail, after an initial live video-presentation. No reason was given by FFT clinicians for not wanting to participate. Participants were eighteen therapists, three supervisors and two team managers. Their experience with FFT ranged from three months to nearly 8 years, with an average of 20 months. Most interviewees were female (*n* = 18). A minority of the participants had pre-pandemic experience with providing VC teletherapy (*n* = 6), mostly providing individual teletherapy (*n* = 4).

#### Intervention

Functional Family Therapy (FFT) is a short and intensive evidence-based model addressing risk and protective factors within and outside of the family and changing family interactions. It is delivered at home rather than a clinic. Within standard FFT, the young person (11–18 years) is referred for behavioural or emotional problems. FFT works through four phases. The *Engagement* phase takes place prior to any session and aims to engage all family members in therapy. During the *Motivation* phase therapists work to decrease hostility, conflict and blame, increase hope and build balanced alliances with all family members. This phase also includes an assessment of the relational functions, i.e., understanding the relational processes in the family. The *Behavioural Change* phase is more active and skill-based. The aim of this phase is to reduce the problems by improving family functioning and developing skills. This will often include cognitive-behavioural strategies. The last phase is *Generalisation*, aimed at extending the improvements to multiple areas and plan for future challenges (Alexander et al., 2013). Multiple randomized controlled trials, dissemination / implementations studies and cost-effectiveness studies have shown FFT to be an effective and cost-effective therapy (Robbins et al., 2016).

This study included five teams from across the South of England. One urban FFT team was part of the National Health Service who provided telephone and VC therapy in the first months of the pandemic using the VC platform Attend Anywhere. In the summer of 2020, they returned to undertaking home-visits in full personal protective equipment. The other four teams were part of FPM, a social enterprise providing FFT in different urban, suburban and rural regions. FFT therapists at FPM continued to provide VC throughout the pandemic, with outdoor visits or home visits being allowed under restricted circumstances. Therapists used different platforms, including Teams, Zoom and WhatsApp video. In the autumn of 2020, FPM introduced Facebook portals, which were provided to families if needed. These devices could be plugged into families’ TVs and included a wide-angle camera that could widen the view to include all members of the family, as well as track family members moving around the room.

#### Analysis

Data analysis was conducted by the first author using inductive thematic analysis within an experiential and contextualist perspective (Clarke, Braun & Hayfield, 2015) in Nvivo 1.4.1. Other investigators were involved in several steps of the process, as described below under ‘validity’.

##### Coding

Text was coded using open coding. The unit of analysis could be a part of a sentence or a couple of sentences together. Data saturation was achieved towards the end of data collection, with no new major themes emerging in the last 5–7 interviews.

##### Development of Themes

After initial coding, themes were identified usin^2006^g the following steps. First, coded text was re-read several times to search for themes (Braun & Clarke, 2006). The identified themes were then reviewed by testing them against the coded text and the full manuscripts to ensure they fitted the data (Braun & Clarke, 2006). Finally, themes were defined and named and the interrelation of the themes was explored in integrative diagrams. These diagrams represent how the themes and codes relate to one another (Urquhart, 2013). To aid in the development of the diagrams, the first author made memos of emerging themes as well as theoretical memos about potential relationships between codes throughout the project (Urquhart, 2013; Robinson, 2011).

##### Reflexivity

The first author took a reflexive stance by being mindful of her own perspective on the topic (namely, that VC could be feasible with many of these families but may be paired with challenges) and cultivating a curious and open stance. A thorough literature review was only conducted after concluding the analyses, to prevent existing theory guiding the interpretation of the transcripts.

##### Validity

Validity of the findings, i.e., reduction of bias, was safeguarded through several processes. Firstly, *coding* was triangulated using investigator triangulation. The second interviewer independently coded seven transcripts. Two of these coded transcripts, as well as the developed codebooks, were compared and discussed between both interviewers to check whether the first author had missed any themes or important codes. Although there were small differences regarding naming of codes or the level of detail of coding, no new codes or themes emerged, thus supporting validity of the codes. Examples of differences in naming are ‘being curious’ versus ‘asking more questions’ and ‘asking parents for support’ versus ‘relying on parents’. An example relating to the level of detail is that one interviewer used two different codes, namely ‘difficulty giving feedback and instructions’ and ‘lack of control over what family is doing/writing’, where the other interviewer had used one more generic code, namely ‘navigating family when doing activities is harder’. Another example is that one interviewer had coded the increased workload separately, whereas the other interviewer had coded this as part of the activities that increased the workload, such as increased between-session contact and increased need for planning and preparation.

*Findings* were triangulated by discussing themes with the second (a quantitative evaluation researcher) and third author (director of one of the participating institutions, qualitative researcher) on multiple occasions. The discussion consisted of a presentation of the emerging themes at several stages to the co-authors, who asked critical questions about the content of the themes and the logic of their relations. These discussions led to refinement of the themes and diagram.

Triangulation was further acquired by using a mixed-method approach (method triangulation) and by interviewing professionals with different roles (data triangulation). Convergence and divergence of the method triangulation is discussed in the Discussion. The aim of the data triangulation was to provide complementary perspectives and so further evaluation of convergence and divergence of data sources was not conducted.

This manuscript describes the results related to the challenges and adaptations of using teletherapy (see Supplementary material for final codebook).

### Results

**Fig. 1:**
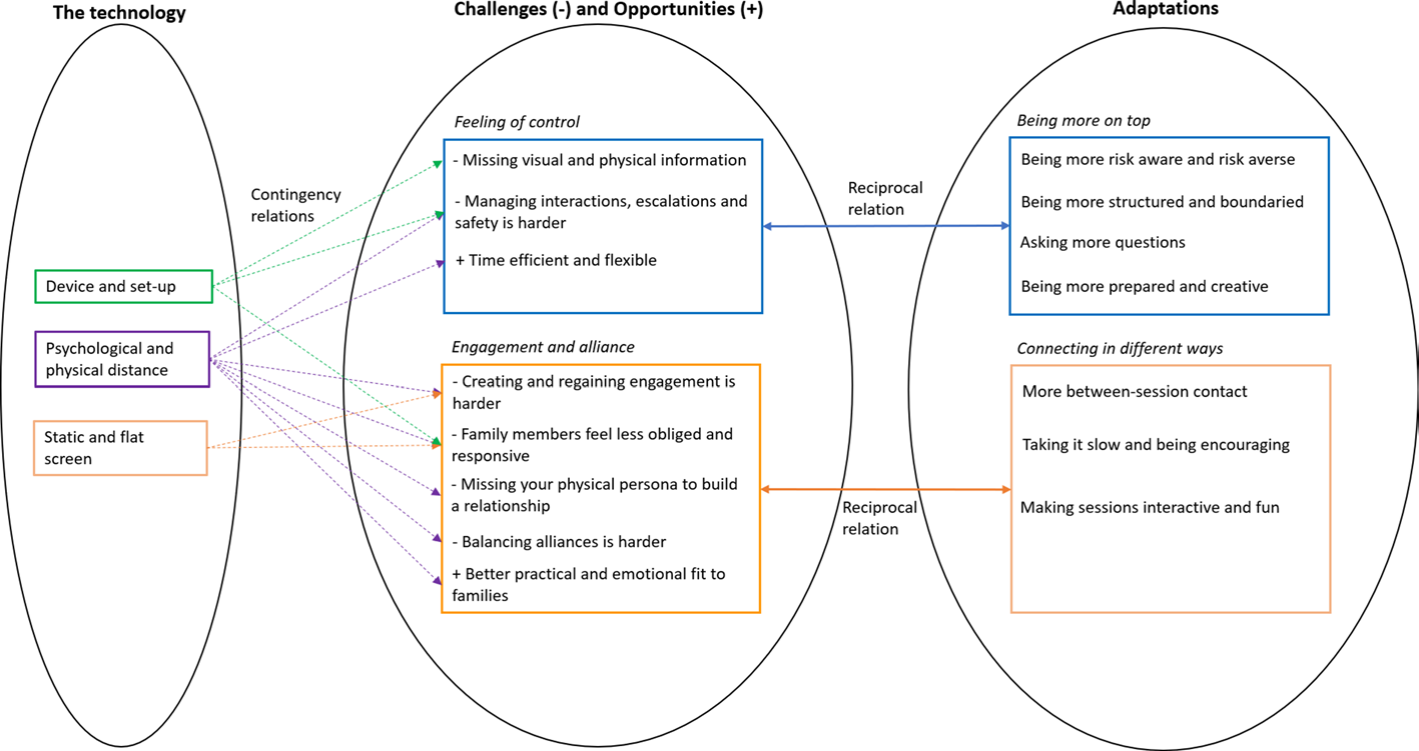
Model representing identified themes and associations of the impact of using videoconferencing in FFT

Based on the interviews with the professionals a model was proposed to provide an initial understanding of how technology impacts upon treatment and what adaptations therapists have made to accommodate these (see Fig. 1). Three technology aspects impacted upon therapy, namely the ‘Device and set-up’, the ‘Physical and psychological distance’ and the ‘Static and flat screen’. Therapists experienced challenges, but also opportunities around two themes, namely a ‘Feeling of control’, including feeling in control of risks and safety, and around ‘Engagement and alliance’. The adaptations mapped onto these two themes. To increase their feeling of control, therapists were ‘More on top’ of therapy. Secondly, they tried ‘Connecting in different ways’. We will explain these themes in more detail below. Quotes are followed by a code, representing the gender (F = female, M = male), the role (T = therapist, S = supervisor, M = manager) and the number of months of experience with FFT of the interviewee being quoted.

#### Technology

##### Device and Set-Up

The available technology mattered. Families often used phones or small screens, meaning that therapists struggled seeing all family members: “Some large families, you’re losing, you can’t see everybody at one time” (MS24). Therapists gave examples of families passing round a phone or holding a phone on a selfie-stick in the air, all of which may not create a comfortable setting for a therapeutic session. About half of the participants mentioned how the introduction of the Facebook portal had made a significant difference: “It is almost like if you can see into the room, and it’s almost like you’re present in the room” (FT11).

Screen freezing and bandwidth drop were common problems and could disturb the flow of therapy, especially if they were experienced on critical moments. They could also demotivate families and impact upon engagement: “It doesn't feel very therapeutic to ask someone to repeat themselves three or four times until your Internet decides it's going to let it through.” (FT36).

##### Physical and Psychological Distance

Teletherapy creates a physical and a psychological distance, both of which created challenges as well as opportunities. The physical distance, mentioned by 12 participants, related to not being present in the same room. The psychological distance or “barrier”, mentioned by almost everyone (*n* = 21), related to lacking “the human touch” and feeling that online could be alienating, awkward or uncomfortable. At the same time, the distance could create a feeling of comfort and safety (*n* = 8).

##### Static and Flat Screen

Half of the participants explained how the two-dimensionality could have a structuring effect, but could also be demotivating and harm engagement of families (see below).

#### Feeling of Control

##### Missing Visual and Physical Information

The first subtheme, mentioned by almost everyone, related to only seeing what was on the screen and potentially missing important visual and physical information about the family, such as how family members reacted to or felt during the session: “Actually, what was quite difficult was gauging their sort of facial responses and expressions. So not sort of knowing how they responded to what I'm saying and what I'm doing, that's quite difficult” (FT5). This was even harder when teenagers didn’t want to be on the camera: “I can't physically see how they responded. I don't know if they have listened to me, hearing me” (FT5). Therapists further struggled gauging “feeling in the room; the tension between people, the small gestures” (MT7).

According to 8 participants, this could result in the motivation phase taking longer or being harder as therapists felt less certain about their assessment of the situation: “It takes longer to be sure. … You can still get that sense of what might be going on, but you can't say that you're pretty sure. You're still always in that: ‘Oh, I think so.’” (FT18). Although one therapist said that he might not actually miss many cues, but was worried that he would.

This restricted view also impacted the assessments of risks and could result in sudden explosions or escalations of emotions (*n* = 18).

FT26: “When you go to people’s houses, is that - not that I judge people’s houses -, but you can sort of tell sometimes how well they're feeling or how not well they're feeling.”

FT18: “You don't truly get a sense of how somebody's presenting, you know. You wouldn't see it necessarily, see a bruise. You wouldn't necessarily see the dad is in the house and he's just fuming upstairs.”

FT11: “If I’m in the room, we can feel it getting hotter or whatever, but if you’re just seeing it on the screen, you might kind of misjudge something.”

##### Managing Interactions, Escalations and Safety is Harder

Almost all participants (*n* = 20) talked about experiencing more difficulties in managing situations in the home due to missing information and not being physically present: “Your presence isn't felt in that room, and so it's quite hard to take charge within a session.” (MT7). Some of these were very practical; therapists needed help from parents to organise sessions and manage the engagement of the children, which could be stressful for parents.

FT5: “Actually what we're asking families to do, is get up a laptop, having to log into Teams, which technically is quite difficult sometimes, having to try find a good position to be on camera, having to all sit there. You rely a lot more on parents sometimes, to be the ones to keep that structure and coordinate and so it is tough.”

FM31: “When you’re in the room with the family, and it’s circus, you can offer a small child a kind of toy to cuddle or a piece of paper and a marker….So there’s a lack of control for the therapist, I think, in having none of that. They completely rely on the parent or parents to try to contain some of that chaos.”

It was also harder to “navigate” families in the activities they needed to do, as explaining activities often required therapists to model things or do things together with the family.

Managing escalations was a big concern for many therapists (*n* = 18). Usually they would interrupt escalations physically, for example by standing up. Online, therapists felt they had less presence and couldn’t use their whole self. Hence, many talked about feeling reduced to a “little squeaky voice” on the screen, or even excluded from the session, because families turned or walked away from the camera or closed the device: “They tend to be able to exclude you on the camera far easier than they can in person” (FT18). If the connection was broken, therapists could no longer check on the safety and the wellbeing of the family and help the family to calm down.

MT7: “They blew up and the young person stormed off and their mum shouting and I can't take control of that situation. And then mum’s hung up. ... I don't know then what is going on in that house. … You think: “Have I just increased the level of risk?” So that young person, I'm not there to being able to do anything about it.”

This created stress for a minority of the therapists (*n* = 5): “Especially when there is a lot of risk involved, I feel really, really afraid, I had to say, when I'm doing it online” (FT3).

##### Time Efficient and Flexible

According to 19 participants, the most important benefit came from the reduced need for travelling. Therapists were used to travelling to visit families in their homes: “I think I save 10 h’ worth of travelling a week” (FT5). This created more time for recording or preparing a session for some (*n* = 5) whilst improving work-life balance for others (*n* = 8): “I like it because I have more of a work-life balance. I think before, travelling to the visits in the locality resulted in more late evening working. …. Whereas now I feel like I can have a bit more of a balance emotionally, myself.” (FT38). According to half of the participants it further allowed more flexible planning of sessions, increasing attendance and clinical effectiveness.

MS24: “If we had a non-attendance, for example, it was much easier to reschedule, because I didn't have to re-travel back out.”

FM31: “If you go into a session with a family, and you’re worried about them, you can say: ‘Hey, can I come back tomorrow morning?’ And you can see them and feel how things are and follow up.”

However, a few therapists (*n* = 3) felt it wasn’t time efficient as working remotely required more time to create engagement (see below), prepare for sessions or drop off resources: “Although there is a traveling time that we're saving, that is more than matched by the need for getting things to people” (FT18).

#### Being More on Top

This lack of control in several domains led therapists to adapt to become ‘Being more on top’. This was done in several ways.

##### Being More Risk Aware and Risk Averse

Most participants (*n* = 18) talked about being more aware of risks, more prepared for risky situations and more proactive in preventing risky situations. Although there was no unified approach, multiple examples were given. For example, one therapist mentioned she would rate the risks during their weekly team meetings higher than when visiting families in-person because of the unknown. Another said she would be quicker with having a family safety plan in place than usual. Several therapists also collaborated more closely with social workers. “I guess it feels even more important, the kind of professional networking, because everybody's getting like a smaller portion of contact with a family. So I guess sharing, putting the pieces together, feels more important, because nobody's really having that much.” (FT4).

Ten participants talked about being more risk averse during sessions, working hard to contain sessions and prevent escalations happening online: “I’m very conservative in how I will have conversations and how I would do things over video” (FT11). This was achieved by interrupting more quickly and using more positivity, for example when reframing behaviour: “I was just more focussed on the positive intention” (FT22). One therapist expressed her concerns that this more conservative approach might lead to disengagement because families might feel they were not doing anything.

##### Being More Structured and Boundaried

Thirteen participants talked about making agreements with families, being more directive and setting clear rules to stay in charge and keep control over situations. “Perhaps the level of structure or the boundaries that need to be put in place, rules if you like for that session, have to be quite clear and defined when we're online” (MS24). For example, therapists made agreements about what to do if the connection would fail, set rules about turn taking or gave instructions on how to sit so everyone could be seen on the screen. It was felt to be important that “we don't lose the thread when we're on camera because it is so easy to be excluded from what's going on in the main assembly” (FT18).

##### Asking More Questions

As therapists missed some visual information, they asked more questions and checked with family members to know how they felt about what was being discussed (*n* = 10). If a teenager didn’t want to be on camera, or if things were happening outside of the screen, therapists needed to collaborate with or rely on parents.

“I just ask what is going on there. So “What is the noise? Who was screaming? Who just left the room? Who just came?” So, basically more general questions to just know what is going on in the room” (FT22).

“I do a lot of just checking in, just, you know, “Are you still with us? What do you think about that?” Or kind of just bringing the conversation back to her [the teenager who is out of view]” (FT36)

##### Being More Prepared and Creative

According to most participants (*n* = 21), sessions needed more preparation, because therapists were restricted in what they could do and how they could manage situations: “A lot more work and thinking and thought has to go into how that session is delivered” (FT7).

During the Behavioural Change phase, sessions would usually be more activity-based to teach new skills to families. Many of these activities, such as games or physical and collaborative activities, were hard to do online. Hence therapists had to rethink their skill-teaching activities (*n* = 13): “Clinically, you have to adapt what skill you're offering to what medium or what hardware they're using” (MS24). Working remotely further added an additional activity to planning these skill-sessions, as they couldn’t take the resources along when visiting the family: “You still have to think about, not just preparing the resources, but how you're going to get them to the family” (FT18). Therapists used different ways of getting the resources to the families, such as dropping off resources, sending resources through the post, sending digital resources or asking parents to gather material from their home. Half of the participants explained how this required organisation and planning, often requiring therapists to plan multiple sessions ahead. According to one third of them, this could be time consuming.

#### Engagement and Alliance

This theme encompasses several challenges around creating engagement and a therapeutic alliance with families. Nevertheless, half of the participants agreed that it was possible to have good relationships online and were often surprised by this: “The thing that surprised me is I formed a bit of a connection, more than I thought I would.” (FT38).

##### Creating and Regaining Engagement is Harder

To create engagement with this hard-to-engage population, therapists were used to being perseverant and visiting families in their homes. Seventeen participants explained how this changed completely due to teletherapy: “I think everyone had to rethink how, what that sort of persistent, assertive thing means on, virtually” (FM31). Phone calls and text messages could be more easily avoided than physical visits at the door.

FS24: “I think online, they've definitely got the option to just press the button to say ‘No’”

FT36: “If a family wasn't so responsive, you might just stop by and leave a note on the door or knock and say “Hi” and introduce yourself.”

Similar issues were experienced around engaging young people, as therapists couldn’t follow the teenager to their room. To regain engagement, therapists needed to rely on parents, asking them to go to the teenager with the device, which parents were not always willing or comfortable to do so (*n* = 8).

FT18: “So there was this argument that was going on outside of my vision, because it was outside of the room. And then mum didn't even get up. She just sat there and she said: “See what I have to put up with?” And I said: “No, I can't see.” I said: “I don't know whether you want to take me out, so I can?” And she said: “No, I'm just going to leave them to it.” And you've got nothing really.”

##### Family Members Feel Less Obliged and Responsive

Even if families were in the session, engagement or motivation levels could be low, according to most participants (*n* = 18). The physical and psychological distance of the therapist made family members feel less obliged to fully participate: “It doesn't feel as serious to other family members” (MT7). Family members could be doing other things at the same time, such as cooking or the wash. “When you're physically there, they’ll treat it more as a session where you all sit in the room together and you will focus” (FT5).

The flatness of the screen further decreased motivation, concentration and responsiveness of families: “I just feel that it's when they sat there, just staring at the screen, is where engagement and motivation is lost” (MT7), which could lead family members to disengage more easily than in-person. Five participants mentioned that, when working with larger families, they struggled to hold everyone’s attention and engagement: “It’s really difficult, because, to hold all of their engagement, have them all at hand, on time, for them to not wander off, to really say you're talking to each of the family members, it can be difficult” (MT11).

##### Missing Your Physical Persona to Build a Relationship

Most participants (*n* = 19) mentioned struggles with building a therapeutic relationship, because they felt they couldn’t use their persona in the same way*: *“You miss what you get and what you feel from a person in the room. You can, when doing, you know what therapists do, you bring a certain level of comfort” (FT36). Part of this was related to the fact that therapists could not show they would be there to support the family in the same way.

MT11: “And it shows less commitment in a way. I mean, you know, they're seeing me make phone calls rather than make regular visits, and they, and that means a lot to, that's quite an expression of care and commitment to be visiting a family a couple of times a week or more to build, you know, to try and gain their trust.”

Eight participants experienced a challenge around matching to families’ interests: “When I walk in the house, I might notice some family photographs of holidays or the way the house is decorated or. All these little things tell me something about them that I can use to build strength-based relational focus.” (MT7). “You may see things in the house, the way they live that you can match to and you can begin to build conversations on.” (FT7).

##### Balancing Alliances is Harder

In FFT it is important to balance alliances and make sure that all family members feel equally heard by and are equally connected to the therapist. According to one third of the participants, this was harder to achieve and maintain online, because of therapist’s missed opportunities for natural, small conversations with individual family members.

FT5*: *“When you're in someone's house and you get glimpses of times, I know it's a whole family approach, but you get glimpses of times where you might get to speak to just one of the young people, because the sibling is in the kitchen or mum’s popped to the loo. … Whereas in a session, it’s all of them all the time and less of those – yes, mum walks away to get the door - but it's not an extent we'd start a conversation with somebody else. So you do miss those little moments where you can pick up on what individual people like or what they're doing.”

Furthermore, the young person often would not want to be on camera, or would even leave the room completely, meaning therapists shared more nonverbal and verbal contact with caregivers than with the teenager (*n* = 14).

FS96: “If her mum and her sister were on the screen, I could see them. It just felt like it was more with them. Even though the young person that was referred was still in the room, it just didn't feel the same and it felt like I was kind of, any kind of bits of banter that we had or the soft stuff, like just smiling at one another and sort of showing empathy and that kind of thing, it didn't feel the same with her, because of not being able to see her.”

FT18: “If a young person walks out of the room and you’re left with mum, do you risk, go: “Oh well, I can’t do the therapy session now” and risk losing mum or do you chat with mum and then risk losing the young person, because she feels ganged up or. So some of that is tricky.”

##### Better Practical and Emotional Fit to Families

Remote working could be a better fit for some families, increasing engagement and the therapeutic alliance. Practically, remote working offered flexibility, allowing the opportunity to plan sessions at more suitable times (e.g. for working families) or to have family members join in from different locations (*n* = 16). Several therapists talked about the benefit for separated families, where teletherapy provided a neutral and comfortable setting, avoiding the necessity to have ex-partners receive therapy in one another’s house for example.

It could also create emotional fit by providing a feeling of comfort or safety, according to most participants (*n* = 19). Therapists cited teenagers joining from their bedroom: “I think there's definitely families where I would have lost the young person had he not been able to go up in his room on his phone instead” (FT24). Joining remotely allowed teenagers to “effectuate a bit of a withdrawal, but still participate” (MT11) and was thereby a way of providing some psychological and emotional distance, comfort and safety. This not only applied to teenagers, but also to some families as a whole, allowing them to “say things a bit more openly than perhaps they wouldn't have done if they were sat next to each other” (FS96), because they could “opt sometimes to angle the phone slightly away” (FT24) or talk to the screen rather than directly to other family members or the therapist. Some families also felt more comfortable not having someone in their house.

#### Connecting in Different Ways

To account for those challenges when engaging families remotely, therapists found several new or additional ways of building connections with families.

##### More Between-Session Contact

Many therapists felt that “you may have to do more video calls before you even begin to scratch the surface of getting to know that person and that person getting to know you” (FT7), therefore increasing their contact with family members through phone, voice notes and text (*n* = 11). Therapists also needed to be flexible and creative with this. For example, by sending letters through the post to younger children without a personal phone.

Besides these virtual contacts, most therapists (*n* = 17) tried to create in-person encounters by doing doorstep visits, “having a quick ‘Hello’”, or dropping off resources as an “excuse to go and see them on the doorstep again, just to kind of keep that relationship going” (FT38)*.* These additional contacts were an opportunity to create engagement and a demonstration of perseverance and willingness to invest in the family (*n* = 10). Therapists were “trying to replace the effort that we would normally make with telephone call effort” (MT11) to show they weren’t going to give up.

Four therapists talked about establishing family WhatsApp groups as an easy way to connect with the whole family at once. More importantly, it also facilitated building balanced alliances as everyone would get the same information in the same way: “There's always a thing with FFT about … ideally not allowing the parent to speak for the young person. For them to have their own [voice], so that was a kind of equalizer” (FM31).

In some situations, face to face sessions were felt to be needed and, when restrictions allowed, outdoor sessions were organised: “I think for this young person in particular, for him to develop trust and to feel comfortable with me, it was important to meet with me in person” (FT3).

##### Taking it Slow and Being Encouraging

Therapists needed to acknowledge and normalise “how it does feel and look awkward” (FT36) and coach families to use teletherapy, both emotionally and sometimes practically, by talking them through the technology step by step (*n* = 13). Therapists felt they’ve “got more of a job to convince people that we can make it work. And so it does take a little bit longer” (MT11). Therapists took it slow and tried “not to go in too heavy” (MT7), taking time to create comfort and build up a relationship (*n* = 10). Where needed, this could include splitting sessions in half, creating shorter and more frequent sessions, as well as being flexible in how family members joined (*n* = 15).

##### Make Sessions Interactive and Fun

To increase engagement during sessions and motivate families to participate, almost all participants (*n* = 21) talked about trying “to find the fun element” (FT24). This was more important online because of the two-dimensionality of the screen. Fun could help to create a better and more engaging therapeutic context: “I found that reframes and point processing and sequencing, it lands better actually in those sessions when they are cooking rather than when you just sat there in that more clinical way just answering questions” (MT7). Making it fun and interactive was especially important when younger children were involved in the sessions. Half of the participants talked about using technology, for example by showing videos or using the blackboard.

### Summary Study A

Study A explored the experiences of FFT professionals with regard to providing FFT using teletherapy during the first nine months of the COVID-19 pandemic. We found that professionals felt less in control when providing teletherapy, which could be especially problematic when dealing with risks and safety around the family. Therapists also struggled with engaging families and building (balanced) alliances. Some participants mentioned this could result in the Engagement and Motivation (EM) Phase taking longer or being more intensive. Nevertheless, therapists proved to be resilient and found innovative ways to adapt to the new circumstances, including strategies to keep a level of control and different ways to connect and engage families. The adaptations of ‘being structured and boundaried’, ‘asking more questions’, and ‘taking it slow and being encouraging’, and the challenge around ‘balancing alliances’ were the least frequently mentioned subthemes (mentioned by one to two third of the participants), whereas all the other subthemes were mentioned by at least 75% of the participants.

## Study B

Study B consisted of analysing routinely collected secondary monitoring data to test several hypotheses which emerged from Study A. Study A provided the following testable findings: (a) we found challenges around engagement and the therapeutic alliance when using teletherapy, (b) professionals consequently mentioned treatment to be longer and requiring more contact or sessions (i.e., more intensive), (c) the challenges may also have increased drop-out, d) flexibility was found to be one of the benefits of VC, which positively impacted attendance of families. Although study A produced additional hypotheses, we did not have any quantitative data available to test these. Given that some of the challenges around alliance, duration and intensity were most pronounced during the EM phase, we specifically evaluate these variables within this phase. We thus hypothesized three negative and one positive effect of using VC:

1. the therapeutic alliance would be lower
2. therapy would be longer or more intensive, during the EM phase (2a) and for the treatment as a whole (2b)
3. drop-out would be higher
4. attendance would be better

Study B also allowed us to evaluate therapeutic outcomes. We could not formulate a hypothesis about this based on Study A.

### Method

#### Participants

We used anonymous routinely collected data from the 286 families who started FFT between March 2020 and April 2021. Nineteen of these families never started therapy and seven families ended prematurely because of non-treatment related reasons (e.g., they moved out of the treatment area). Families that were still in treatment by the end of April 2021 were only included if they had completed the EM Phase. This resulted in a final sample of 209 families. However, not all families could be included in all analyses, as will be detailed below.

The majority of the families had closed therapy (*n* = 134, 64%), either because they had completed treatment (*n* = 82, 61%), or because they dropped out (including re-referral and out-of-home placement of the referred adolescent; *n* = 52, 39%). The remainder of the families were still in treatment (*n* = 75, 36%).

Most families (79%) consisted of two to four family members, with a maximum of seven. Half of the families (56%) included at least one sibling. Almost all families were referred by child welfare (95%). The most frequent referral reasons were verbal conflict within the family (36%), family violence (27%) and delinquent behaviour or gang involvement (11%). Adolescents mostly lived with one caregiver (53%) or two caregivers (40%), with a minority living with extended family members (6%) or in another situation (1%). Almost half of the referred adolescents were female (46%) and the average age was 14.00 years old (*SD* = 2.26).

#### Measures

All measures were part of the routinely collected monitoring data of FFT. These measures were chosen as they were available retrospectively for the whole research period. We had to rely on retrospective data, as no one had foreseen the COVID-19 pandemic. The monitoring data allowed us to include data from the very beginning of the pandemic.

The measures have been developed as part of the quality assurance system of FFT. To ensure high response rates on a continuous basis, these have been developed to be simple and short to provide clinicians with ongoing feedback on their delivery of FFT. No information on validity was available for these measures.

##### Teletherapy

Case file information was used to extract the percentage of sessions that had been received in-person or remotely. Families were subsequently categorised into the following three groups: (1) mostly in-person (≤ 10% telesessions), received by 24% of the families, (2) mixed (11–89% telesessions), received by 43% of the families, and (3) mainly remote therapy (≥ 90% telesessions), received by 33% of the families. Two families had only cancelled sessions and between-session contacts, so we could not calculate the percentage of teletherapy received by these families.

##### Therapeutic Alliance

Alliance was measured using the Family Self Report (FSR), a 7-item questionnaire using a 7-point Likert scale to assess hopefulness and alliance (1 = I strongly disagree / not at all; 7 = I strongly approve / a lot’). We used the four items measuring therapeutic alliance, namely ‘Overall, how much do you approve or disapprove of the way your therapist is treating your family?’, ‘Whether or not you agree with the way your therapist is treating your family, how much do you like your therapist’, ‘How much do you trust your therapist?’ and ‘How much do you feel your therapist trusts and likes you?’. Cronbach’s alpha of this scale was good (α = 0.81). The FSR was completed by all family members individually during the first two sessions of the Motivation Phase. We calculated an average per assessment. The two assessments conducted during the EM phase were averaged per respondent to represent the overall alliance during EM. We also calculated a split alliance. Similar to previous work, a split alliance was operationalised as a difference in alliance between two family members (young person and caregiver) that was larger than 1 *SD* unit, resulting in a dichotomous variable.

##### Drop-Out

This was extracted from the case file and was defined as any family who did not complete all the phases of FFT for any reason.

##### Treatment Duration and Treatment Intensity

This was extracted from the case file. Intensity was operationalised in two different ways: a) the number of between-session contacts and b) the number of sessions. Duration and intensity were calculated for the whole treatment period, but also for the EM phase only. This was because the hypothesis was that duration and intensity would be impacted by the challenges around engagement and alliance, which is most important during the EM phase.

##### Attendance

Attendance was not calculated at participant level, but at session level. Sessions were scored as 'attended’ (in person, video or phone), ‘cancelled’ or ‘no show’.

##### Outcomes

The outcomes were measured using the Therapist Outcome Measure (TOM), a therapist-rated outcome measure completed at the end of treatment. It consists of twelve items on a 6-point Likert scale (0 = things are worse; 1 = no change; 2 = little better, 3 = some better; 4 = lot better; 5 = very much better or ‘not applicable’). The questions relate to several domains on which improvements could have occurred, including the adolescents’ behaviour, parenting skills, communication skills in the family, family conflict, school attendance and performance, and alcohol and drug use of the adolescent. Cronbach’s alpha of this scale was excellent (α = 0.98). The average was used in the analyses.

#### Analyses

##### Assumptions and Missing Data

Assumptions of the tests were checked, and outliers were winsorized to two standard deviations above the mean. There were eight missing values for duration of EM phase due to errors. For the alliance variables, response rate was 40% for the young people and 48% for the caregivers. Families with no information on alliance had a significantly younger referred child, were more likely to have dropped out, had received less sessions and between-session contacts, had a shorter treatment duration and worse outcomes. Other demographic variables were not significantly different for families with and without completed alliance assessments. Most importantly, the use of teletherapy was not related to non-response on alliance, suggesting we can reliably use this measure.

##### Comparison of Groups

To evaluate whether teletherapy groups (mainly in-person, mixed, mainly remote) were comparable, we compared families in either group on demographics using ANOVA’s and chi-square tests. Any demographic variable that significantly differed between the three teletherapy groups, was included as covariate in subsequent analyses.

##### Main Analyses

The effect of teletherapy on the therapeutic alliance, treatment duration, intensity, drop-out and outcome was analysed using three MANCOVA’s. The independent variable was teletherapy group membership (mainly in-person, mixed and mainly remote). For the first two MANCOVA’s (Hypothesis 1 and 2), grouping was based on the portion of remote therapy during the EM phase only (based on the whole sample, *n* = 209). Due to missing data on alliance, the MANCOVA for alliance only included 76 families. For the third MANCOVA, grouping was based on the whole treatment period, thus only including families who had closed therapy (drop-out and completion, *n* = 132). We used Wilks’ Lambda, unless group sizes and variances varied significantly. In that case, we referred to Pillai’s criterion as a multivariate test statistic.

To test *Hypothesis 1 (alliance)*, we conducted a MANCOVA on the whole sample with alliance (alliance of young person and alliance of caregiver) as the dependent variable (*n* = 76). As split alliance was a dichotomous variable, this outcome variable was analysed using a chi-square test.

To test *Hypothesis 2a (duration and intensity EM phase)*, we conducted a separate MANCOVA with duration and intensity of the EM phase (number of between-session contacts during EM, number of sessions during EM and duration of EM phase) as dependent variables (*n* = 201). This hypothesis was tested separately from Hypothesis 1 as only a portion of the families completed the alliance assessments. Conducting a separate MANCOVA thus allowed to include a larger sample for testing Hypothesis 2.

To test *Hypothesis 2b (duration and intensity whole treatment) and evaluate outcomes,* we conducted a MANCOVA on the subsample of families who had closed treatment (drop-out and completion, *n* = 132) with the number of between-session contacts, number of sessions, treatment duration and therapist-reported treatment outcome as dependent variables.

*Hypothesis 3 (drop-out)* was analysed using a chi-square test as drop-out was dichotomous.

*Hypothesis 4 (attendance)* was tested using a chi-square test on all 2592 planned sessions to identify which sessions (remote through video, remote through audio or in-person) were least likely to be cancelled or be a ‘no show’.

### Results

Table 1 and 2 present correlations between all variables. Tables 3, 4 and 5 present means and standard deviations for all variables, split by teletherapy group.

**Table 1:** Correlation table for full sample (*N* = 207)

	1	2	3	4	5	6	7	8
1. Percentage telesessions during EM	–							
2. Family size	− 0.17*	–						
3. Alliance young person during EM	0.04	− 0.17	–					
4. Alliance caregiver during EM	− 0.16	− 0.14	0.28*	–				
5. Split alliance during EM (0 = no)	− 0.16	0.08	− 0.38**	− 0.04	–			
6. Number of between–session contacts during EM	− 0.16*	0.12	0.00	− 0.11	0.29*	–		
7. Number of sessions during EM	− 0.02	0.24**	− 0.11	− 0.11	0.21	0.07	–	
8. Duration of EM phase	− 0.10	0.11	− 0.14	− 0.20	0.32**	0.41**	0.37**	–

*p < 0.05, **p < 0.01

**Table 2:** Correlation table for subsample of closed treatments (*n* = 132)

	1	2	3	4	5	6	7
1. Percentage telesessions	–						
2. Family size	− 0.11	–					
3. Dropout (0 = completed)	− 0.08	− 0.11	–				
4. Number of between-session contacts	− 0.24**	0.09	− 0.19*	–			
5. Number of sessions	− 0.02	0.25*	− 0.81**	0.26**	–		
6. Length of treatment	− 0.06	0.24**	− 0.67**	0.30**	0.75**	–	
7. Average treatment outcome	0.04	0.10	− 0.75**	0.24**	0.69**	0.55**	–

*p < 0.05 **p < 0.01

**Table 3:** Means and standard deviations for alliance, split by teletherapy group (*n* = 76)

	Mainly in-person	Mixed	Mainly remote
	n = 26	n = 18	n = 32
Alliance young person	5.58 (1.37)	5.79 (0.96)	5.76 (0.83)
Alliance caregiver	6.15 (0.83)	6.42 (0.62)	5.97 (0.79)
Split alliance (yes)	46%	61%	28%

Groups significantly different from in-person therapy, as tested with the MANCOVA or chi-square, are printed in bold

**Table 4:** Means and standard deviations for duration and intensity during EM phase, split by teletherapy group (*n* = 201)

	Mainly in-person during EM phase	Mixed during EM phase	Mainly remote during EM phase
	n = 87	n = 40	n = 74
Number of between-session contacts during EM phase	7.75 (7.17)	8.65 (9.07)	**4.24 (5.88)**
Number of sessions during EM phase	3.32 (1.22)	**4.05 (1.18)**	3.24 (1.38)
Length of EM phase (days)	30.40 (28.22)	**41.18 (33.77)**	23.28 (20.76)

Groups significantly different from in-person therapy, as tested with the MANCOVA or chi-square, are printed in bold

**Table 5:** Means and standard deviations for duration, intensity, outcomes and drop-out for closed cases, split by teletherapy group (*n* = 132)

	Mainly in-person	Mixed	Mainly remote
	n = 37	n = 57	n = 38
Number of between-session contacts	19.65 (18.33)	18.53 (17.82)	10.37 (15.76)
Number of sessions	8.08 (6.56)	**13.70 (5.18)**	7.82 (5.79)
Length of treatment (days)	92.19 (52.40)	**137.54 (36.93)**	89.82 (58.27)
Therapist-rated outcome	2.20 (1.34)	**2.87 (1.08)**	2.42 (1.22)
Drop-out (yes)	62%	**16%**	47%

Groups significantly different from in-person therapy, as tested with the MANCOVA or chi-square, are printed in bold

#### Comparison of Groups

Larger families were significantly more likely to receive in-person therapy than remote therapy (*F*(2) = 4.36, *p* = 0.01; *M*_in-person_ = 3.88, *M*_mixed_ = 3.60 and *M*_remote_ = 3.20). There were no other differences between families who received mainly remote, mixed or in-person therapy on the demographic variables.

#### Hypothesis 1 (Alliance)

The multivariate test for comparing the level of alliance between teletherapy groups was not significant (*F*(4) = 1.25, *p* = 0.29), meaning that the use of teletherapy during the EM phase did not predict alliance of the young person (*p* = 0.90) or caregiver (*p* = 0.10), controlling for family size. Split alliance did also not differ between groups (χ^2^(2) = 5.41, *p* = 0.07). See Table 1 for the correlations and Table 3 for the means per group.

#### Hypothesis 2 (Duration and Intensity EM Phase)

The multivariate test for testing duration and intensity during the EM phase showed that, controlling for family size, teletherapy was a significant predictor (*F*(6) = 3.82, *p* < 0.01, *ƞ*^2^ = 0.06). Univariate results showed that teletherapy related to all outcomes (*p* < 0.01, *ƞ*^2^ = 0.5–0.7 for all outcomes). Contrast results showed that families receiving mainly remote therapy during EM had less between-session contacts than families receiving mainly in-person (see Table 1). Families receiving a mix had significantly more sessions than families receiving in-person as well as a significantly longer EM phase (see Table 4).

#### Hypothesis 2 (Duration and Intensity Whole Treatment) and Evaluation of Outcomes

We also conducted a MANCOVA comparing duration, intensity and therapeutic outcome between teletherapy groups for families who completed FFT, controlling for family size. Teletherapy was a significant predictor according to the omnibus test [*F*(8) = 5.77, *p* < 0.00, *ƞ*^2^ = 0.16]. Univariate results showed an effect of teletherapy on all outcomes except between-session contact (*p* < 0.00, *ƞ*^2^ = 0.20–0.22 for number of sessions and treatment length; *p* = 0.02, *ƞ*^2^ = 0.06 for treatment outcomes). Contrast results showed the mixed group had significantly more sessions, a longer length of treatment and better outcomes than the in-person group. See Table 2 for the correlations and Table 5 for the means per group.

#### Hypothesis 3 (Drop-Out)

Drop-out was significantly related to using teletherapy [χ^2^(2) = 22.55, *p* < 0.00] with the mixed group having the lowest drop-out rate. See Table 2 for the correlations and Table 5 for the means per group.

#### Hypothesis 4 (Attendance)

Sessions were least likely to be cancelled or have a no show if they were over video (8%), more likely to have a cancellation or no show in-person (19%) and most likely if they were over the phone (39%; *χ*^2^ (2) = 171.37, *p* = 0.000).

### Summary Study B

Study B tested hypotheses generated by findings from Study A. Specifically, it was hypothesised that teletherapy would be related to weaker therapeutic alliances, more drop-out and more intensive treatment (longer or more contact between sessions), but also better attendance. We found that sessions were less likely to be cancelled when using videoconferencing (but not when using a phone) compared to in-person. There was no relation between alliance and teletherapy. Evidence for the hypothesis about treatment intensity was mixed. Although the mixed group had more sessions and a longer treatment period (during EM and in total) than the in-person group, this was not the case for the families receiving mainly remote therapy. Moreover, we found an opposite effect with remote families having less contact between sessions than families receiving mainly in-person therapy. The mixed group also had lower drop-out and better therapist rated treatment outcomes than families receiving in-person therapy. The findings regarding treatment intensity for the mixed group could relate to the lower drop-out rate, as early drop-outs generally have had a shorter therapy with fewer sessions.

## General Discussion

This study aimed to explore the feasibility of providing remote FFT. More specifically, it aimed to (1) determine if the delivery of FFT online was adversely affected through this method of delivery, (2) to understand barriers and adaptations to the online delivery of FFT, and (3) to understand if a fully online or blended delivery of FFT (partially online) is good enough to become an acceptable model of delivery post pandemic. In response to the first aim, we did not find any indication that teletherapy would be less effective than in-person therapy. On the contrary, we found that a mix of remote and in-person was related to lower drop-out rates and better outcomes. Moreover, attendance was better at video sessions than at in-person sessions. Nevertheless, in response to the second aim, we did find that therapists experienced significant challenges (i.e., feeling less in control and experiences more challenges around engaging and relating to families) and found innovative ways of addressing these challenges (i.e., by being more on top and connecting in different ways). In response to the third aim, we therefore conclude that providing family therapy through VC to families on the edge of care seems possible, but that some in-person contact may be required in some instances. We will discuss the details in more detail below, as well as how they relate to other findings from the literature.

Our quantitative results regarding the therapeutic alliance corroborate recent research into Multisystemic Therapy that the alliance with families on the edge of care does not seem to be compromised by using teletherapy (Lange et al., 2021). However, many professionals mentioned challenges around building engagement and a therapeutic alliance when using VC. We therefore must bear in mind that these positive findings are likely due to the hard work, creativity and perseverance of professionals. As one of the therapists stated “I'd like to think that the quality [of the relationship] is still really good, because we've all worked really hard to adapt our sessions” (FT8).

Similarly, therapist-rated outcomes and drop-out rates were not affected by using teletherapy and were even better for families receiving a mix of in-person and remote therapy. The results for treatment intensity were mixed. Families receiving mainly remote therapy were comparable to families receiving mainly in-person therapy although they did have fewer between-session contacts during the EM phase. Yet receiving a mix of remote and in-person was related to more intensive treatment than mainly in-person therapy. Previous research on FFT did not find any difference regarding treatment length and number of sessions between 2019 and 2020 (Robbins & Midouhas, 2021).

Several of the adaptations required closer collaboration with parents to manage the situation in the home, from set-up to making sure all family members are attending and engaging. Although this corresponds with previous research around teletherapy with children (Crum & Comer, 2016; Maier et al., 2021; McLean et al., 2021), therapists in this study mainly talked in terms of the need to ‘rely’ on parents, as it could be problematic to request parents to collaborate when parents didn’t feel capable or willing to support the therapist. These families often experience problems within family functioning, communication, parent–child relationships and parenting behaviour (Deković & Bodden, 2019). In such situations it may be challenging, even counter intuitive, having to rely on parents. This information enriches previous findings regarding liaising with parents when working with children (Crum & Comer, 2016; Maier et al., 2021; McLean et al., 2021) and points to some of the challenges around this collaboration with certain types of families.

The reduced feeling of control was an important challenge to many therapists, especially around assessing and managing risks and conflicts online. This shows that heated emotions are not solely the realm of couple therapy (Hardy et al., 2021; Springer et al., 2020), but equally arise when providing family therapy. There was no unified approach to assess risks, which suggests that more work may be needed to develop effective strategies for assessing risks when being online. We found that therapists managed risks by being more conservative and risk averse, which corroborates previous findings (e.g., McLean et al., 2021). More research is needed to understand how this different approach towards conflicts affects effectiveness of interventions.

Another unique challenge to family teletherapy was balancing alliances. We did not find any previous study investigating balancing alliances when providing teletherapy, even though it is essential in any relational therapy to prevent split alliances (Friedlander et al., 2018; Muñiz de la Peña et al., 2009). In the current study, therapists reported that they struggled to balance alliances when using teletherapy. However, split alliances were not more common when using teletherapy according to the monitoring data. This suggests that therapists have been able to balance alliance even when facing this new technological environment.

Although reporting difficulties around engagement and alliance is not unique to this study (Mc Kenny et al., 2021; McLean et al., 2021; Springer et al., 2020), our study highlighted a unique challenge for this population of hard-to-engage families. Previous research has compared teletherapy to office-based therapy. In such situations, attending teletherapy requires less effort from families than in-person therapy. Families in our study, however, would normally be visited in their own home. Engaging families without in-home sessions proved challenging and many therapists complemented telesessions with in-person contacts.

Some of the adaptations were similar to previous studies. For example, therapists regained some feeling of control by being more prepared, asking more questions, setting clear rules and boundaries, making agreements, especially around conflict situations, and being more risk averse (McLean et al., 2021; Springer et al., 2020; Taylor et al., 2021; Wrape & McGinn, 2018). However, other adaptations have not been previously documented, such as using WhatsApp family groups to ensure balanced alliances and closer collaboration with other professionals around the family to assess risks. The introduction of the Facebook portals, which allowed for a wider-angled view, also proved to be a beneficial adaptation. According to therapists, it increased their feeling of control and facilitated building engagement and alliance. Yet, current guidelines provide very little guidance on which devices or platforms could be more or less suitable (McLean et al., 2021).

### Clinical and Research Implications

This study demonstrates promise for the future of family teletherapy as it suggests that systemic family teletherapy is feasible and can achieve comparable outcomes and therapeutic alliances, even with families who may be more difficult to engage. If future research confirms these findings, teletherapy could prove useful for improving reach and accessibility of evidence-based interventions, for example for families in remote areas.

Nevertheless, further developments of providing family therapy through VC to families on the edge of care would require careful consideration of a number of topics. Firstly, clinicians would need to consider how and when to involve or not involve parents. By not being in the same room, clinicians have less control over the situation and need to rely more on parents to get participation from the young person or information on what is going on. This can, however, cause problematic situations. Clinicians must be aware of this tension and different scenarios or solutions should be discussed with colleagues or in supervision meetings. Another area needing further clinical exploration is risk management when using VC. Clinicians should discuss different options for assessing risks through VC.

Research is needed to understand whether using a more conservative and risk averse approach during VC sessions negatively impacts effectiveness. Research is also needed to compare the feasibility and effectiveness of fully remote approaches with hybrid approaches. The current results suggest that some in-person contact may be crucial for certain families to create engagement. The current study was inconclusive regarding the need for an increase in between-session contacts, session frequency or therapy duration to create engagement and positive outcomes. Future research should explore this.

Policy makers and providers of care should consider investing in appropriate devices as we have found that this can have a major impact on the feasibility of successfully using VC.

### Strengths and Limitations

This study has several limitations. Firstly, our study was contextually bound by the COVID-19 pandemic and findings may be different if assessments were conducted during other times. For example, the alliance during teletherapy may have been scored higher than in a non-pandemic situation, because families appreciated the effort of the therapist and the challenges they faced. Therefore, we need well-designed trials with a control group to investigate the feasibility, acceptability and effectiveness of family teletherapy. Also, families were not randomly assigned to teletherapy or in-person treatment. Rather, the national restrictions combined with personal circumstances of therapist and family guided which families received in-person or teletherapy. Therefore, our findings about the effect of teletherapy on the therapeutic alliance, drop-out or outcome must be interpreted with caution. More rigorous, preferably randomised, designs are needed in future to fully test the effect of teletherapy on outcomes. It is also important to note that this study focussed on FFT as an example of systemic family therapy and that little information on the diversity of the sample was available. Also, we included only two providers of FFT, all in the UK. We believe that many of the challenges and adaptations mentioned in this study are applicable to other systemic family interventions, in the UK as well as abroad, and this is supported by similarities found with previous research. We must nevertheless bear in mind that some findings may be unique to FFT or this specific sample and may not generalise to other settings, populations or intervention programmes. Lastly, we did not include the perspective of the families when conducting the interviews nor when evaluating the outcomes. Given the specific challenges this study has found regarding family teletherapy, more research needs to be conducted to understand the experiences of parents, children and young people in these situations and how this impacts on client-rated outcomes.

This study also has several strengths, such as the mixed-method approach and the large sample size in both studies. It further studied an underrepresented population in the field of teletherapy. Therefore, this study is of high clinical relevance, providing both quantitative evidence on feasibility as well as a qualitative understanding of the potential challenges and adaptations of providing remote systemic family therapy.

### Conclusion

This study is one of the first studies to explore the feasibility of systemic family teletherapy with families on the edge of care. We conclude that systemic family teletherapy seems feasible and promising. This offers the potential for the further testing of hybrid interventions which combine in-person and online working so that the benefits may accrue to the family, the therapist and potentially service commissioners, especially in more rural and remote areas where in-person therapy is prohibitively expensive. This study also highlights some important challenges as well as potential adaptations for providing systemic family teletherapy, especially around the role of parents in managing situations, managing conflicts and risks, using appropriate devices and engaging hard-to-engage families when not visiting in-person. This study provides a valuable starting point for further thinking about providing such hybrid or fully online systemic family teletherapy.

## Supplementary Information

Below is the link to the electronic supplementary material.

Supplementary file1 (DOCX 20 kb)

## Data Availability

The data supporting the quantitative findings is openly accessible at the Open Science Framework (https://osf.io/h3yrp/, doi: https://osf.io/h3yrp/). The first author takes responsibility for integrity of the qualitative and quantitative data and accuracy of the data analyses.

## Code Availability

The codebook for study 1 is included as supplementary material. The syntax used for the analyses of study 2 can be requested from the authors.
