# Supplementary material for: The Feasibility of Providing Remote Functional Family Therapy with Adolescents During the COVID-19 Pandemic: A Mixed-Method Study
Source: Child Youth Care Forum. 2022 May 2;52(2):441–66. doi: 10.1007/s10566-022-09692-y (PMC9060401; doi:10.1007/s10566-022-09692-y)
Supplement: Supplementary file 1 — Supplementary file1 (DOCX 20 kb) [file 10566_2022_9692_MOESM1_ESM.docx]

**Supplementary materials: Interview Format**

1. Can you tell me how the FFT treatment has been impacted by the COVID-19 pandemic and the transition to online therapy?

*What is the most important difference according to you?*

**Delivery of FFT**

FFT has had to move online. I would like to ask a couple of questions about your experiences with online or remote FFT (through videoconferencing).

1. Has the transition to online therapy affected the way in which you engage with families prior to the first session?

*Can you explain how? What have you learned? What were the challenges, opportunities? What skills, adaptations, innovations have you made? What surprised or disappointed you?*

1. Has the transition to online therapy affected the way in which you motivate families?

*Can you explain how? What have you learned? What were the challenges, opportunities? What skills, adaptations, innovations have you made? What surprised or disappointed you?*

1. Has the transition to online therapy affected the way in which you realise behavioural change and then move into generalisation in families?

*Can you explain? What have you learned? What were the challenges, opportunities? What skills, adaptations, innovations have you made? What surprised or disappointed you?*

**Risks**

1. Has the transition to online therapy affected the way in which you asses and address risks in families?

*Can you explain? What have you learned? Have you experienced that your assessment of the risks changed when a family moved to from online to in-person or vice versa? What were the challenges, opportunities? What skills, adaptations, innovations have you made? What surprised or disappointed you?*

**Therapist wellbeing**

1. How has the COVID-19 pandemic affected you personally and professionally?

*How has this changed over the past months (since the beginning of the pandemic)?* *How has this impacted your work with your families?*

**Fit to families and professionals**

1. Does online FFT work better or worse for some families than others?

*What are the characteristics of these families / For what type of families does it work better or worse?*

1. Just as it may suit some families better than others, online working may also suit some therapists better than others. How does online therapy work for you? How does it match your way of working?

*Can all therapists learn to provide FFT online?* *What characteristics or skills do therapists need to work online successfully? Has your attitude or confidence regarding online working changed? Has this impacted your work with the families?*

1. You probably have worked with families that have experienced both online and face to face therapy. Can you tell me a bit about your experiences in these situations?
   1. When families transitioned from face to face to online?
   2. When families transitioned from online to face to face?
   3. When families received a hybrid form of treatment (including both online and face to face elements)?

**Use of VC in future**

1. Would you continue to deliver FFT online in the future if that was possible (but not mandatory)? If so, under what kind of conditions?
   1. Would it need to be hybrid in some way?
   2. Would you do this with all families or only with certain type of families or certain problems?
   3. Would there be any situations you would *not* want to use online means?
2. What support would you need to continue to deliver FFT online in the future, in material and/or in knowledge?

*What work on FFT needs to be done to deliver it successfully through video? For people who left the organisaton: What support would you need if you would provide online systemic therapy in the future?*

**Concluding**

1. Is there anything else you want to say or add? Anything important that we haven’t discussed yet?

**Supplementary materials: Codebook**

| **Technology** | **Challenges and opportunities** | **Adaptations** |
| --- | --- | --- |
| **Device and set-up** | **Feeling of control** | **Being more on top** |
| - Devices, platform and set-up | Managing interactions, escalations and safety | Asking more questions |
| - Home set-up family | - Managing escalations and safety | Being more prepared and creative |
| - Not everybody fits on a screen | - Harder to ensure safety | - Adapting to new context |
| - Benefits of portal | - Harder to interrupt and divert | - Usual activities and resources are not available |
| - Technical failures | - Risk of being excluded | - Being creative and thinking out of the box |
| **Psychological and physical distance** | - Stressful and anxious regarding risks | - More mental preparation for sessions |
| - Awkward and uncomfortable | - Managing interactions and setting | - More planning and prepare during BC phase |
| - Creates comfort and safe context | - Harder to navigate families | - Time consuming |
| - Creates disengagement | - Need to rely on parents to manage situations | Being more risk aware and risk averse |
| - Missing human and physical element, alienating | Missing physical and visual information | - Being more on top of risks (risk aware) |
| **Static and flat screen** | - Harder to assess feeling in the room and interactions between family members | - Collaboration with SW, other professionals |
|  | - Harder to assess risks and gauge the situation | - Working hard to prevent escalations (risk averse) |
|  | - Missing nonverbal cues and visual information | Being more structured and boundaried |
|  | Time efficient and flexible | - Being directive and assertive |
|  | - Reduced travel time creates efficiency and flexibility | - Being structured, focused and concise |
|  | - Better work-life balance | - Making rules and agreements with family |
|  | - Less exhausting |  |
|  | - More clinical time |  |
|  | - More flexibilty for planning of sessions |  |
|  | - Not time efficient |  |
|  | **Engagement and alliance** | **Connecting differently** |
|  | Balancing alliances is harder | Making sessions interactive and fun |
|  | - YP is screenshy | - By using creativity and humour |
|  | Better practical and emotional fit to families | - By using technology (eg videos and whiteboard) |
|  | - Distance creates comfort and safety | - To counter static screen |
|  | - Allows for remote engagement, engaged withdrawal | - To create therapeutic context |
|  | - Fit to autonomous families | - To engage younger children |
|  | - Less direct and reduces anxiety | More between-session contact |
|  | - Less intrusive in house | - Demonstrating perseverance and willingness |
|  | - Practical fit | - Being reliable and regular |
|  | - Allows to join remotely | - Doorstep or outdoor visits (in-person encounters) |
|  | - Practical fit to family life | - Dropping or sending resources |
|  | - Building strong alliance online is possible | - Establishing family WhatsApp group |
|  | Creating and regaining engagement is harder | - Increased phone and text contact |
|  | - Creating engagement harder | Taking it slow and being encouraging |
|  | - Need to rely on parents | - Create comfort, take it slow |
|  | - Restricted with regard to engagement strategies | - Engagement phase longer |
|  | Family members feel less obliged and responsive | - Motivation phase longer |
|  | - Clients less obliged and more distracted | - Shorter (and more frequent) sessions |
|  | - Disengaged during the session, distractions | - Support family emotionally (coaching and acknowledging medium) |
|  | - Leaving the room more easily | - Support family practically |
|  | - Engaging multiple members on screen harder |  |
|  | - Less concentration, motivation and responsiveness |  |
|  | - Getting them to practice in BC |  |
|  | Missing your physical persona to build a relationship |  |
|  | - Less able to use therapeutic person to build relationship |  |
|  | - Matching is harder |  |
|  | - Showing trustworthiness and willingness to support is harder |  |
